# Defective Nuclear Lamina in Aneuploidy and Carcinogenesis

**DOI:** 10.3389/fonc.2018.00529

**Published:** 2018-11-20

**Authors:** Elizabeth R. Smith, Callinice D. Capo-chichi, Xiang-Xi Xu

**Affiliations:** ^1^Department of Cell Biology, Sylvester Comprehensive Cancer Center, University of Miami Miller School of Medicine, Miami, FL, United States; ^2^Laboratory of Biochemistry and Molecular Biology, Institute of Biomedical Sciences, University of Abomey-Calavi, Abomey Calavi, Benin

**Keywords:** nuclear envelope, nuclear lamina, nuclear deformation, nuclear morphology, nuclear budding, lamin A/C, aneuploidy, ovarian cancer

## Abstract

Aneuploidy, loss or gain of whole chromosomes, is a prominent feature of carcinomas, and is generally considered to play an important role in the initiation and progression of cancer. In high-grade serous ovarian cancer, the only common gene aberration is the p53 point mutation, though extensive genomic perturbation is common due to severe aneuploidy, which presents as a deviant karyotype. Several mechanisms for the development of aneuploidy in cancer cells have been recognized, including chromosomal non-disjunction during mitosis, centrosome amplification, and more recently, nuclear envelope rupture at interphase. Many cancer types including ovarian cancer have lost or reduced expression of Lamin A/C, a structural component of the lamina matrix that underlies the nuclear envelope in differentiated cells. Several recent studies suggest that a nuclear lamina defect caused by the loss or reduction of Lamin A/C leads to failure in cytokinesis and formation of tetraploid cells, transient nuclear envelope rupture, and formation of nuclear protrusions and micronuclei during the cell cycle gap phase. Thus, loss and reduction of Lamin A/C underlies the two common features of cancer—aberrations in nuclear morphology and aneuploidy. We discuss here and emphasize the newly recognized mechanism of chromosomal instability due to the rupture of a defective nuclear lamina, which may account for the rapid genomic changes in carcinogenesis.

## Introduction

The Cancer Atlas Project (1) determined that TP53 is the only common genetic mutation (96%) found in high-grade serous epithelial ovarian cancer (2, 3), the most common histological subtype of the gynecological malignancy. Nevertheless, extensive study in culture cells and model organisms indicate that TP53 mutations alone are unlikely sufficient for ovarian carcinogenesis, and additional factors and events are required. In studies using mouse models, merely inactivation of p53 by gene deletion in ovarian epithelial cells is insufficient for epithelial tumorigenesis (4, 5), and even following transplantation into wild type mice to allow an extended lifespan and thus longer rearing time, granulosa rather than epithelial tumors develop in the p53 mutant ovaries (6). Thus, the genetic changes required for the development of epithelial ovarian cancer, particularly the high-grade serous type, are not yet satisfactorily understood.

Other common genomic changes in ovarian carcinomas revealed by the cancer genomic study are extensive aneuploidy and gene copy number aberrations (1). Over 100 years ago, Boveri first recognized the connection between an abnormal number of chromosomes and neoplasms (7, 8). Aneuploidy as a result of chromosomal numerical instability is a prominent feature of carcinomas, and is often assumed to play an important role in initiation and progression of cancer (9–13). Although overwhelming evidence is lacking, chromosomal instability and thus aneuploidy are commonly thought to propel genomic evolution, and to shape a genome that presents as a malignant phenotype (9–16).

A prevalent view is that chromosome instability and aneuploidy provide wide phenotypic heterogeneities in cancer cells and lead to rapid selection of clones with chromosomal compositions that have growth and survival advantages (17, 11). Enabling cancer initiating cells with the plasticity of gene copy number, aneuploidy/chromosomal instability likely plays important roles in clonal selection during cancer initiation, progression, and possibly resistance to therapeutic drugs. The importance and urgency in understanding the mechanism(s) of chromosomal instability and causes of aneuploidy are evident, and are essential to uncover key factors and events in the development of high grade serous ovarian carcinomas.

Generally, aneuploidy is thought to result from mitotic errors and chromosomal non-disjunction during mitosis (18–24). However, recent observations suggest that a nuclear envelope defect may cause chromosomal numerical instability and aneuploidy in cancer (25–28), and nuclear budding leading to the loss of chromosomes at the cell cycle interphase may be a major mechanism in the development of aneuploidy in ovarian carcinogenesis (26). The current review will emphasize this newly recognized mechanism in generating aneuploidy due to a defective nuclear envelope, which may be an under-appreciated pathway in carcinogenesis.

## Aneuploidy and chromosomal numerical instability in cancer initiation and progression

Like many common solid tumor types, ovarian cancer of primary tissues or derived cell lines is characterized by aneuploidy, or an abnormal and unbalanced number of chromosomes compared to normal diploid cells. Most human ovarian cancer cells possess a hyperdiploid (>46) to subtetraploid (< 96) chromosome number (29). The increase in chromosome number over normal cells accounts for the larger nuclear size of ovarian cancer (30). To reach such a chromosomal number, one possibility is that the diploid precursor cells progressively acquire chromosomes in a shift up manner. Another route is the formation of tetraploid intermediates following a progressive loss of individual chromosome through formation of micronuclei (15, 16, 20, 31). Additionally, centrosome amplification and multipolar mitotic division of polyploidy cells may also produce aneuploid cancer cells (32, 33). These proposed routes to a cancer karyotype have been assessed and studied experimentally (26, 34–36).

The commonly accepted doctrine of a carcinogenic pathway does not account for the prevalence of aneuploidy in human cancer (37, 38). However, its prevalence in human cancer cells suggests an important role for aneuploidy in the development of human cancer (9–13).

Nevertheless, polyploidy and aneuploidy usually are unfavorable for cell growth, and can be detrimental for cell survival (39–44). Inactivation of TP53 seems to be able to overcome the growth impairment of aneuploid cells (45–47), and p53 is regarded as the guardian of chromosomal number and genome (48–50). Thus, p53 mutation may not itself induce aneuploidy, but rather allow survival and growth of aneuploid cells. The hippo tumor suppressor pathway is also activated upon genomic perturbation (51). Survivin is another survival factor of aneuploid cells independent of p53 inactivation (52).

Laboratory experiments have attempted to assess the importance of aneuploidy in carcinogenesis. Cultured cells can be transformed i*n vitro* to tumorigenic lines by mutations and deletion of individual oncogenes, and tumor suppressor genes without the need for chromosomal instability (53), and mouse models based on engineered oncogenic mutations often develop tumors of normal ploidy (54). In contrast, it was reported that drug-induced cytokinesis failure generates tetraploids and subsequent aneuploids that promote tumorigenesis in p53-null mammary epithelial cells (34) and mouse ovarian epithelial cells (26, 35). Aneuploid cells were identified in ovarian cysts, suggesting the development of aneuploidy may be an early event in carcinogenesis (55).

## Mechanisms for the generation of chromosomal numerical instability and aneuploidy in carcinogenesis

Although a correlation between aneuploidy and malignancy has been recognized and the significance speculated, the causes of aneuploidy in cancer remain unsettled at a mechanistic level (10, 12, 13).

**Figure 1:**
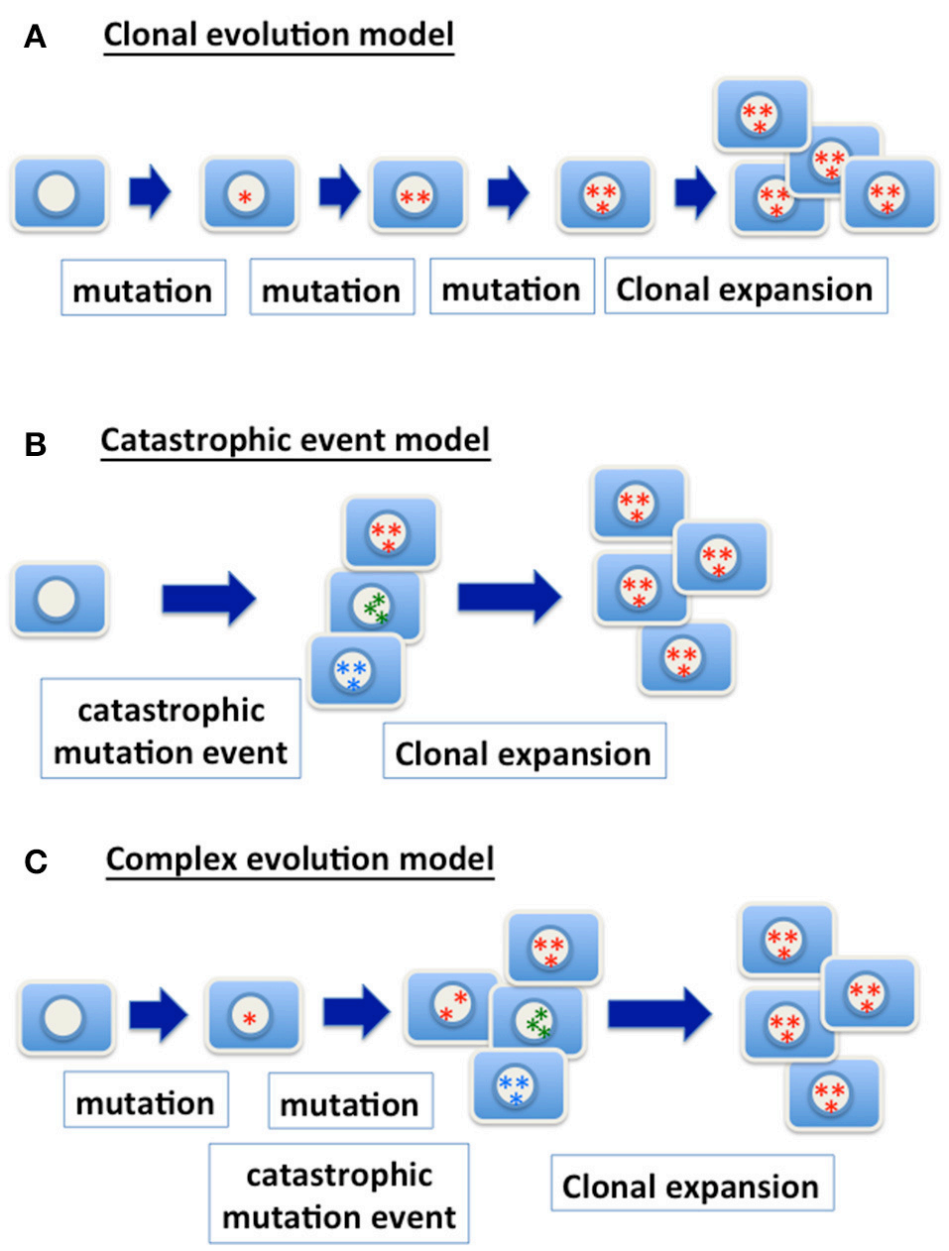
Models of genetic changes in carcinogenesis**. (A)** Clonal evolution model: the traditional model of clonal evolution in cancer development suggests that precursor cells gradually gain relevant genetic mutations (gene point mutation, deletion, amplification, chromosomal gain and loss, etc.) in tumor progression. The sequential addition of each mutational event enhances clonal growth and selection, and the clone with the most mutations expands into a tumor mass and presents the malignant phenotype. **(B)** Catastrophic event model: a catastrophic genetic event triggers massive chromosomal re-arrangement, or gain or loss of multiple chromosomes. Most of cells with such catastrophic genetic changes likely will be purged. However, rare clones may survive and be further selected and expanded, and ultimately present a malignant phenotype. **(C)** Complex evolution model: likely the mechanism in achieving the genetic changes in cancer is much more complex, and one scenario is the combination of multiple mechanisms. Considering a model combining accumulation of mutation and catastrophic event, progenitor cells with a relevant point mutation (such as TP53) are expanded to form a precursor lesion. A catastrophic genetic event enables the generation of cells with a spectrum of genomic variety. Ultimately, clones with an optimal chromosomal composition and genetic changes expand and present a malignant phenotype.

Paralleling the model in which sequential and progressive genetic changes in the form of gene mutations lead to oncogenesis (37, 38), cells undergoing transformation may also gradually gain chromosome numbers over multiple clonal expansions to achieve a hyperdiploid (>46) to subtetraploid (< 96) state (Figure 1A). However, the usually complex karyotypes of cancer cells (29) unlikely can be achieved simply by one or multiple chromosomal unbalanced segregations. Rather, the chromosome profiles appear to arise from a tetraploid intermediate with additional multiple reductions of single chromosomes during multiple rounds of mitotic events.

Based on genomic profiling of cancer, the complex karyotypes in some cancers may be the result of a single (or a few) catastrophic event (56, 57) (Figure 1B). Extensive changes in chromosome number during nuclear budding in interphase may achieve massive assembling of chromosomes in cancer cells that have a defective nuclear envelope. Likely, mutations and gradual clonal evolution, as well as massive chromosomal changes from a single catastrophic event, contribute to the heterozygous and complex genomic landscape of malignant cells (Figure 1C).

The development of aneuploidy by chromosome mis-segregation is generally considered a main cause of chromosomal instability in cancer (18–22). Centrosome amplification is observed in some cancer, and is another possible mechanism for the development of aneuploidy (32, 33). Mitosis in the presence of three or more centrosomes will certainly divide the chromosomes into an aneuploid state. Tetraploid cells are believed to form often following mitotic failure, and aneuploid cells are produced from the tetraploid intermediates in subsequent mitotic events (15, 16, 20, 31, 58).

The studies of chromosome segregation in mitosis have yielded impressive understanding of the genes and mechanics during cytokinesis. The naturally occurring intermittent errors during cytokinesis result in chromosome non-disjunction and subsequent unbalanced chromosomal distribution (18–22). Mutation in genes involved in cytokinesis, such as Mad2, CenE, etc., can cause an increased chromosomal segregation error and result in predisposition to tumorigenesis (15). However, mutations in these genes are not common in cancer (1).

In recent years, transient rupture of nuclear envelope of cancer cells in interphase is observed (59, 60) and considered an under-appreciated mechanism of chromosomal instability (25–28). Indeed, some experiments from our lab support that a structural defect in the nuclear envelope caused by the loss of one or more nuclear envelope structural proteins may be a major cause of chromosomal numerical instability and aneuploidy in ovarian cancer cells (26). A defective nuclear envelope in cancer progenitor cells as a result of loss or reduced of nuclear lamina structural proteins Lamin A/C may lead to the rapid chromosomal and karyotype changes (26). The weakened nuclear envelope can undergo transient rupture in interphase (59, 60), leading to rapid chromosomal changes and deviant karyotypes as seen in human ovarian cancer (25–28).

## Nuclear envelope defects in cancer cells

Enlarged and deformed nuclei are characteristics of cancer cells, and the aberrant nuclear morphology universally correlates with malignancy (30, 61–63). In the clinical setting, the shape and size of nuclei are used as diagnostic and prognostic indicators, referred to as “nuclear grade” (64, 65). Based mainly on the nuclear morphology of cells sampled, the PAP test (or PAP smear), developed by Dr. Papanicolaou in the 1930s, distinguishes between benign and malignant cells to make a pathology assessment of uterine and cervical cancers (66).

In the last five decades, many investigators have attempted to decipher the mechanisms responsible for deformed and enlarged nuclei of cancer cells (47, 67–71). An early proposal was that the altered cellular DNA and chromatin of a cell in a diseased state might cause the changes in nuclear shape through the physical contacts with the nuclear envelope (72, 73). Alternatively, changes in the nuclear matrix and/or nuclear envelope proteins have been postulated, and deformation of nuclear morphology was shown to associate with oncogenic signaling (67, 69, 70), but no definitive conclusions have been established regarding the molecular basis of nuclear deformation in malignant cells or its mechanistic link with the properties of malignancy (63, 68).

The nuclear envelope structural proteins, Lamin A/C, are absent in around 60% of ovarian carcinomas, and show heterogeneous staining in about 30% of these, though as controls the proteins are present in ovarian surface epithelia, surrounding the nuclei (47). Based on high throughput analyses to identify aberrantly expressed proteins, one study reported increased Lamin A/C expression in ovarian cancer (74). However, the proteomic study used normal ovarian tissues (mostly stromal instead of ovarian surface epithelia) for comparison, which are not proper controls. In histochemical analysis, the ovarian epithelial cells of the surface layer were found to be highly positive, whereas the stromal cells were largely negative for Lamin A/C (74). Thus, the correct interpretation of the result should be that 39% of ovarian cancer cases are positive for Lamin A/C, and Lamin A/C proteins are lost or greatly reduced in 61% of ovarian cancers (74). Another report identified Lamin C to be reduced/lost in malignant ovarian cancer but not in borderline tumors, based on results from 2-dimensional gel electrophoresis (75). Thus, the previous studies generally support the finding that Lamin A/C proteins are lost in about 60% of ovarian cancer (47).

Examined by immunofluorescence staining, all the primary ovarian epithelial cells have strong expression of Lamin A/C and show a smooth and oval-shaped nuclear morphology (47). Lamin A/C is lost or greatly reduced in most ovarian cancer cell lines, or the expression is heterogeneous in the cell population. Results from Northern and qRT-PCR analyses found that Lamin A/C mRNA is often present despite the loss of the proteins (47). It is now known that increased AKT and cell cycle-associated phosphorylation of Lamin A/C lead to protein degradation (76–78). Although not yet explicitly investigated and established, phosphorylation in interphase and subsequent protein degradation is a likely main cause of low and lost Lamin A/C proteins in ovarian cancer.

Suppression of Lamin A/C proteins with siRNA oligonucleotides results in extensive aberrations in nuclear morphology, from 30 to 60%, based on several experiments (47). If the Lamin A/C-suppressed cells were followed by time-lapse video microscopy, nearly all cells show nuclear deformation at some point during a 6-h time course as the cells move around. Thus, a low Lamin A/C protein level likely accounts for a deformed nuclear morphology seen in ovarian cancer cells. The loss of Emerin following the elimination of GATA6 in ovarian cancer also accounts for the deformation of nuclear envelope (71). Likely, defects or loss in additional nuclear envelope structural proteins may account for some other cases of nuclear deformation in cancer (79). Thus, loss or reduction of one or more nuclear envelope proteins (including Lamin A/C, Emerin, Nesprins) may account for a deformed nuclear morphology of malignant cells.

## Biology of the nuclear lamina: lamin A/C

Lamin A/C expression is minimal in embryonic stem cells and early embryos, and is progressively increased in nearly all tissues in later developmental stages (80, 81). The initiation of Lamin A/C expression is associated with cell differentiation, suggesting that Lamin A/C expression may serve as a limit on the plasticity of cells for further developmental events (82–84). Additionally, the cell types that seem to lack Lamin A/C, such as embryonic carcinoma cells and some cells of the spleen, thymus, bone marrow, and intestine in the adult mouse may fall into the “stem cell” category, but the general correlation will need to be carefully tested.

Lamin A/C mutations cause several human diseases known as laminopathies, including muscular dystrophy, lipodystrophy, and progeria (85–90). Loss of Lamin A/C expression is often found in both blood cancer cells (91, 92), and solid tumors including those of breast (79, 93), colon (94, 95), gastric (96, 97), lung (98), prostate (70), and ovarian (47).

The cell biology of the nuclear envelope has been well-studied in human cells and model organisms (99–102). Lamin A/C, but not Lamin B1, is critical for the maintenance of a smooth and oval shaped nucleus (103). Mutations or loss-of-function in several nuclear envelope structure proteins, including lamin, emerin, Man1, and Baf in *C. elegans*, cause similar nuclear and mitotic phenotypes such as an enlarged and deformed nucleus, defective chromosome segregation, and the formation of chromatin bridges between divided nuclei, suggesting a critical role for lamin and other nuclear envelope proteins in cytokinesis and mitosis (104, 105, 106).

Lamin A/C null mice die at 4–6 weeks of age due to cardiac degeneration, a phenotype mirroring muscular dystrophy in humans (86). In cellular studies, Lamin A was found to be required for the stability of the Rb protein (107) and to regulate the MAPK pathway (108). Additionally, mutations in Lamin A/C interfere with mitosis and cell cycle progression in mammalian cells (89, 109). These findings are consistent with roles for these nuclear envelope proteins in both maintaining the nuclear structure and mediating cytokinesis/mitosis across species. Furthermore, Lamin A plays roles in chromatin organization (99, 110, 111). The roles of Lamin A/C in signaling, mitosis, and chromatin regulation are suggested to account for laminopathies, and also the cells with defective nuclear envelope proteins are inclined to genomic instability due to aberrant gene expression and chromosomal numerical instability (87, 112, 113).

Unique recurrent *de novo* point mutations in LMNA gene in human were discovered to be the cause of Hutchinson–Gilford progeria, a servile premature aging syndrome (114). The mutations lead to the production of an altered Lamin A protein, known as progerin that lacks a protease cleavage site and is not properly processed (115). Unable to remove the farnesylated C terminal, progerin accumulates on nuclear lamina, leading to nuclear envelope deformation, delayed nuclear envelope disassembly in mitosis, cell cycle defect, chromosomal instability, and premature senescence (89, 109, 112, 88, 115). Intervention with compounds and small molecule inhibitors that increase proliferative and delayed senescence in cells derived from LMNA mutant progeria patients associates with rescue of nuclear blebbing and deformation (116), suggesting a defective nuclear envelope in the causes of the pathology.

The analyses in cells, invertebrates, and mammals all suggest that Lamin or Lamin A/C plays important roles in cell functions that can affect cell differentiation, proliferation, and chromosomal instability (90, 102, 104, 106, 113).

## Conserved roles of nuclear envelope and lamina in mitosis

In model organism *C. elegans* that has only one lamin gene, loss of Lamin (104) as well as other nuclear envelope structural proteins (MAN1 and emerin) leads to mitotic failure, polyploidy, micronuclei, and chromatin bridges (105). For mammals, there are three lamin genes (101), and unlike that in *C. elegans*, mammalian lamin genes are not essential for cell division and development (84, 86).

Nevertheless, in ovarian cancer cells as well as primary human ovarian surface epithelial cells in which Lamin A/C expression is suppressed, frequent mitotic failure, and regression of mitotic furrows were observed (26). This observation is consistent with a role of lamin in cytokinesis in worms and flies in which the only lamin gene is essential for mitosis (104). Lamin A/C is not required for cytokinesis in mammalian cells but affects the cell cycle (86, 89, 109). A likely explanation is that the redundancy of the three lamin genes in mammals compensates for the requirement in mitosis when one lamin gene is absent, but the cells lacking Lamin A/C may present a higher frequency of mitotic failure and cell cycle defects. As Lamin A/C plays a role in forming new nuclear envelopes to enclose the chromosomes of the daughter cells, its absence may make the process less efficient and increase the frequency of mitotic failure. Indeed, an increased mitotic failure and the formation of tetraploid nuclei were observed in Lamin A/C suppressed cells (26, 47).

The Lamin A/C-suppressed cells also have higher frequency of tripolar division, presumably from polyploid cells that have more than two centrosomes (26, 47). The multipolar mitosis of polyploidy cells presumably should result in aneuploid cells. Aneuploid cells with unbalanced gene dosage are growth retarded and undergo cell growth arrest or death (40, 44, 117). A p53 mutation allows the aneuploid cells to survive and undergo clonal selection (45, 47). Even with the inactivation of TP53, most aneuploid cells generated from transient loss of Lamin A/C likely perish afterward, but ultimately, a population of cells with a unique chromosomal composition and TP53 mutation is selected and expanded to form cancer (26, 47). Thus, the experimental results advocate a concept that a deformed nuclear envelope is the main source of chromosomal instability of the cancer cells, and is the cause rather than a consequence of neoplastic transformation (26).

## Consequences of lamin A/C loss in cancer: mitotic failure, transient nuclear envelope rupture, and nuclear budding

Studies and prior consideration overwhelmingly focus on the development of aneuploidy from chromosome non-disjunction during mitosis (18–22). The reduction of chromosome number by the formation of micronuclei is largely considered a result of lagging chromosome(s) during mitosis (18, 118). However, the breaking off of nuclear materials by “nuclear budding” to form micronuclei at the interphases has been also observed (26, 119, 120), particularly by using time lapse imaging and GFP-histone H2B to monitor nuclear content (121, 122). Presumably, the breaking off of nuclear materials and subsequent formation of micronuclei also lead to aneuploidy, though the commonality of nuclear budding at gap phases is not certain.

A recent publication detailed the observation of frequent transient nuclear envelope rupture during interphase in human cancer cells (59). In addition, it has also been shown that micronuclei formed in cancer cells often undergo irreversible splintering from the nucleus, resulting in the loss of one or more chromosome(s) (60). Migrating cancer cells are especially vulnerable to nuclear envelope rupture as a result of physical force exerted on the nuclear envelope in the process of moving the nucleus (123). Presumably such events produce chromosomal instability and may be an overlooked mechanism in cancer genomic instability (25, 27, 28). Additionally, the formation of micronuclei may cause chromatin breakage and clustered chromosomal rearrangements, a phenomenon known as “chromothripsis” (124–126). Such impacts on both chromosome number and structure may explain the massive genomic changes found in ovarian carcinomas.

Consistent with such observations, experiments demonstrated that cells with suppressed Lamin A/C expression show frequent nuclear budding (26). In the Lamin A/C-suppressed ovarian epithelial cells, narrow protrusions/herniations of nuclear materials monitored by GFP-histone H2B often break off from the main nuclear body to form micronuclei, which gradually fade away, leading to aneuploidy (26). The Lamin A/C-suppressed cells are stunted in growth, presumably due to aneuploidy, and cell proliferation can be restored by the loss of p53 (47). In the experiments, the formation of aneuploid cells was confirmed by karyotyping (26). Because of the high prevalence of aneuploidy after Lamin A/C-suppression, it has been suggested that a nuclear envelope defect, resulting from loss or severe reduction of Lamin/C proteins, underlies the main cause of aneuploidy in cancer (26).

**Figure 2:**
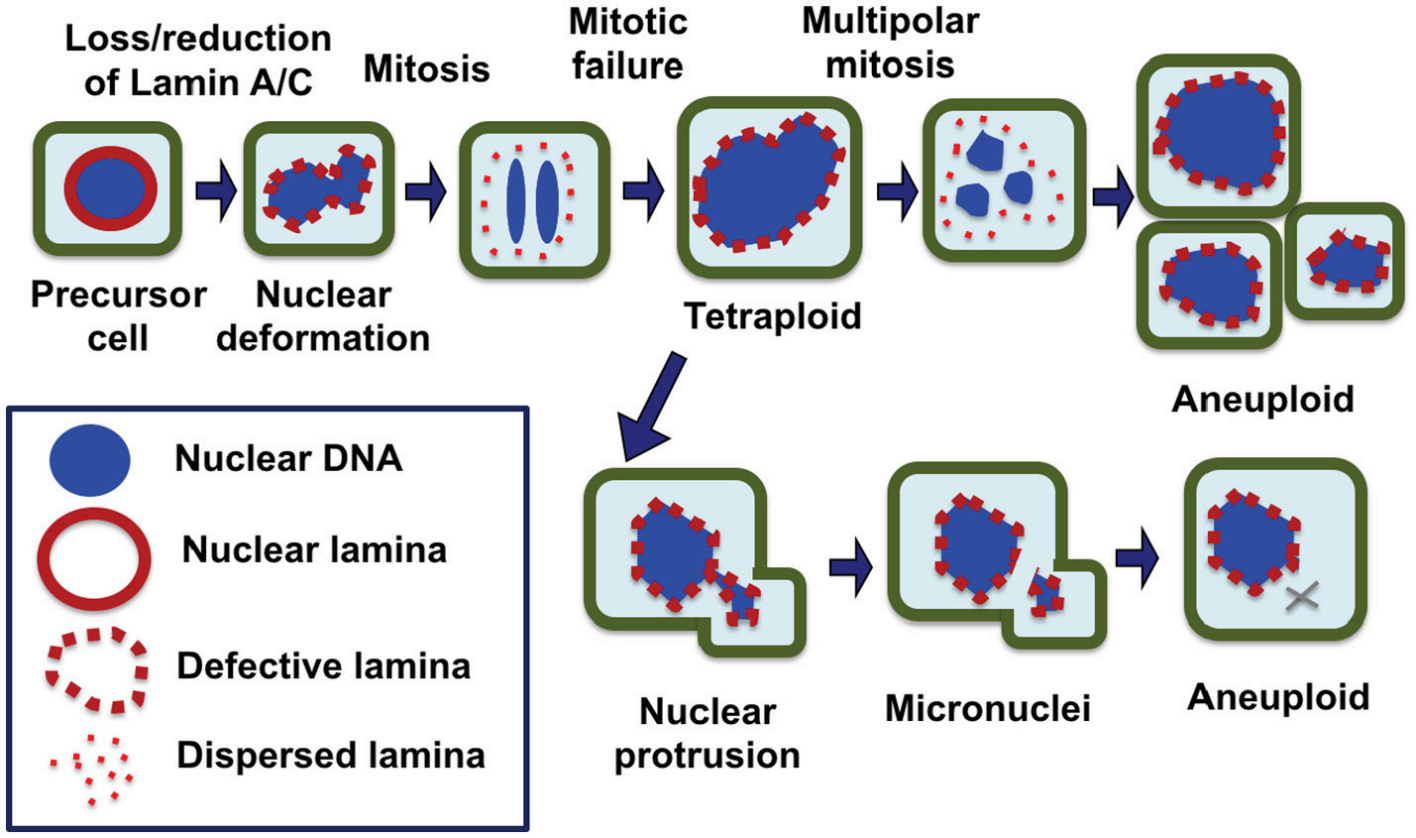
Nuclear envelope defects responsible for the generation of aneuploid cells in carcinogenesis. We propose that a defective nuclear envelope structure as a result of loss of a nuclear lamina component such as Lamin A/C is the main cause of chromosomal numerical instability and aneuploidy in cancer. Loss of Lamin A/C expression is common in ovarian cancer, which results in a misshapen nucleus in malignant cells. Furthermore, Lamin A/C plays an important role in the formation of a new nuclear envelope in daughter cells at the completion of mitosis. In the absence or reduction of Lamin A/C, the cells have higher failure in completing cytokinesis and the dividing nuclei fuse back to form a tetraploid cell, a likely intermediate. Aberrant multipolar division from the tetraploid intermediates can generate aneuploidy cells. Additionally, Lamin A/C deficient cells frequently undergo nuclear budding at interphase, and nuclear protrusions can break off to form micronuclei, which undergoes collapse and in closed chromosome(s) is lost. As a result of catastrophic mitotic events and gradual chromosome losses, the cells with an optimal chromosomal profile may gain growth advantage and be selected to expand into tumor mass.

Thus, we propose that the formation of micronuclei by nuclear budding because of a deformed and malleable nuclear envelope may be the main mechanism in chromosomal instability and the development of aneuploidy in cancer cells, especially those have lost/reduced the nuclear lamina protein, Lamin A/C (Figure 2). Possibly, chromosome DNA string breakage during the budding of micronuclei contributes to chromosomal structural alterations commonly observed in solid tumors, in addition to aneuploidy (124, 125). In this model, we also suggest that the loss/reduction of Lamin A/C leads to frequent failure in mitosis, resulting the formation of tetraploid cells, a likely intermediate to aneuploidy. The polyploidy cells undergo increased frequency of nuclear budding and also undertake frequent multipolar divisions to generate aneuploidy cells (26) (Figure 2).

## Conclusions and remarks: a nuclear envelope defect causes aneuploidy and cancer development

Past studies on the mechanism of the generation of aneuploidy mainly focused on chromosomal non-disjunction (127). The idea that a nuclear envelope structural defect causes chromosomal instability and aneuploidy in cancer has not been sufficiently investigated, but recently this notion has been contemplated (25–28). Based on many of these recent studies and commentaries, as well as our preliminary findings, and we suggest that a nuclear envelope structural defect, such as the loss or reduction of Lamin A/C, a structural protein of the nuclear lamina, may lead to aneuploidy by both mitotic failure and subsequent formation of tetraploid intermediates, and the loss of chromosome(s) through nuclear budding at interphase and the generation and consequent rupture of micronuclei (Figure 2). We suggest that the loss of the nuclear envelope structural proteins, Lamin A/C, may underlie these two hallmarks of cancer: nuclear envelope defects and chromosomal instability.

No doubt chromosomal non-disjunction during cytokinesis contributes to aneuploidy of cancer cells. However, we suggest that a nuclear defect (loss of Lamin A/C proteins) is the common (more than 80% of cancers show nuclear deformation) cause of chromosomal instability and aneuploidy. Defective nuclear envelopes and lamina result in aneuploidy and epigenetic dysregulation, leading to cell clonal evolution to generate malignant cells. This may be a fundamental mechanism for the development of ovarian cancer, and we postulate that loss of Lamin A/C and TP53 mutation are synergistic and sufficient for the development of aneuploid and malignant ovarian cancer (26, 47). Although mainly based on the consideration of ovarian cancer, the conclusion likely can be applied to solid tumors in general, as neoplastic cells of many cancer types show both a deformed nuclear morphology and aneuploidy.

## Author contributions

CC performed the majority of works cited from the authors' lab. X-XX prepared the first draft of the review article, and ERS made extensive revision and editing. All three authors discussed and agreed with the concepts developed and content used.

### Conflict of interest statement

The authors declare that the research was conducted in the absence of any commercial or financial relationships that could be construed as a potential conflict of interest. The reviewer AW and the handling Editor declared their shared affiliation.
